# Computational Model-Assisted Development of a Nonenzymatic Fluorescent Glucose-Sensing Assay

**DOI:** 10.1021/acssensors.4c02117

**Published:** 2024-11-13

**Authors:** Lydia Colvin, Diana Al Husseini, Dandan Tu, Darin Dunlap, Tyler Lalonde, Muhammed Üçüncü, Alicia Megia-Fernandez, Mark Bradley, Wenshe Liu, Melissa A. Grunlan, Gerard L. Coté

**Affiliations:** a Department of Biomedical Engineering, Texas A&M University, College Station, Texas 77843, United States; b Center for Remote Health Technologies and Systems, Texas A&M Engineering Experiment Station, College Station, Texas 77843, United States; c Department of Chemistry, Texas A&M University, College Station, Texas 77843, United States; d School of Chemistry, University of Edinburgh, Edinburgh EH9 3FJ, U.K.; e Department of Electrical and Computer Engineering, Texas A&M University, College Station, Texas 77843, United States

**Keywords:** biosensor, glucose sensing, fluorescence anisotropy, competitive binding, mannose, concanavalin A

## Abstract

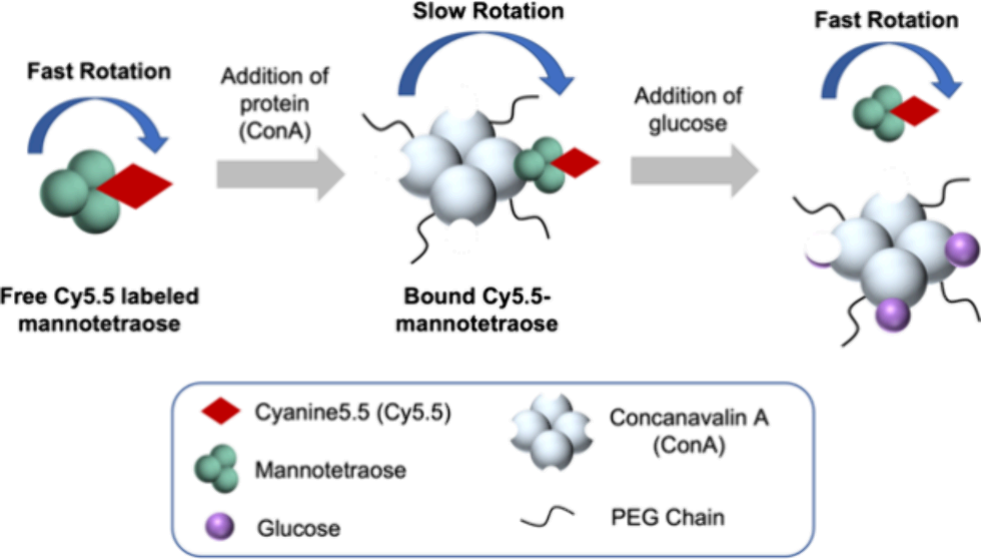

Deep-red fluorescence was implemented in this fully injectable, nonenzymatic glucose biosensor design to allow for better light penetration through the skin, particularly for darker skin tones. In this work, a novel method was developed to synthesize Cy5.5 labeled mannose conjugates (Cy5.5-mannobiose, Cy5.5-mannotriose, and Cy5.5-mannotetraose) to act as the fluorescent competing ligand in a competitive binding assay with the protein Concanavalin A acting as the recognition molecule. Using fluorescence anisotropy (FA) data, a computational model was developed to determine optimal concentration ratios of the assay components to allow for sensitive glucose measurements within the physiological range. The model was experimentally validated by measuring the glucose response via FA of the three Cy5.5-labeled mannose conjugates synthesized with Cy5.5-mannotetraose demonstrating the most sensitive response to glucose across the physiological range. The developed method may be broadly applied to a vast range of commercially available fluorescent dyes and opens up opportunities for glucose measurements using nonenzymatic assays.

Research advancements in the field of continuous glucose monitoring for diabetes management have focused on creating glucose biosensors that have competitive accuracy and sensitivity, a fast response time, are cost-effective, cause minimal discomfort, require less frequent sensor replacements, and hence can enhance the overall experience and quality of life for the patient with diabetes.^1^ As of 2021, the International Diabetes Federation (IDF) has estimated that 1 in 10 adults globally have diabetes, equivalent to an astonishing 537 million people.^2^ The increasing prevalence of diabetes has been a driving factor to designing and creating alternatives to the standard finger-prick method for self-monitoring of blood glucose (SMBG) because of its inconvenience, painfulness, and single-point-in-time readings.^3^ The serious short- and long-term consequences of poor blood sugar management has prompted the development of continuous glucose monitors (CGMs).^4^

For the past two decades, electrochemical indwelling CGMs have dominated the commercial market including companies such as Abbott, Medtronic, and Dexcom allowing measurements every 1 to 5 min and hypo/hyperglycemic alarms.^5^ However, these systems do require replacement of transcutaneous sensors every 5 to 14 days. Moreover, the sensors can damage the skin and must be secured with adhesive tape, which may result in the development of a rash or dislodgement. Although rare, they also pose a risk of infection due to their transcutaneous nature.^3,5^ These electrochemical CGMs rely on the enzyme glucose oxidase, which utilizes the glucose present to facilitate a redox reaction to produce gluconolactone and hydrogen peroxide. Traditionally, the sensor electrochemically measures the amount of the hydrogen peroxide (often via an electrocatalyst), which is related to the concentration of glucose^3,6^ (although more recently via electrochemical analysis of reduced flavin adenine dinucleotide, FADH_2_). This detection method demands a direct physical connection between the subcutaneously embedded sensor and the device adhered to the outer surface of the skin. In addition, these enzymatic glucose sensors have inherent limitations, such as variance due to dissolved oxygen levels, decreases in the catalytic activity of the enzyme from the immobilization process and biofouling, limited reproducibility, and low stability during prolonged operation.^7^

Based on a nonenzymatic, optical-based mechanism, the Senseonics Eversense E3 CGM is a fully subcutaneous sensor with FDA approval for up to 180 days of continuous use.^8^ The sensor contains a diboronic acid-modified hydrogel that encases core electronics and optics including a LED and two photodiodes.^9^ This CGM represents the introduction of fluorescence-based glucose biosensors with the potential for longer lifetimes. It utilizes changes in fluorescence intensity of a polymer upon glucose binding to the bis-boronate moieties that disrupt photoinduced electron transfer (PET) fluorescence.^10^ The fluorescence of the anthracene boronic acid sensor is in the ultraviolet (UV) or blue region.^11^ Owing to the epidermis serving as a protective barrier for underlying tissues to damaging UV radiation, external illumination at these wavelengths is precluded. To ensure the light’s interaction with the sensing chemistry, the LED excitation source is built-in into the implant. However, housing of electronic or optical components within the implant itself leads to a larger-size (Ø 3.5 × 18.3 mm) and hence the requirement for surgical implantation via incision in the upper arm. A bulky transmitter is also required to be affixed to the skin over the implanted sensor. Possible system failures by the optical hydrogel sensor, electronics, optics, or anti-inflammatory drug-eluting silicone collar result in a burdensome and costly replacement of the sensor.

A fluorescence-based biosensor that would enable a smaller, injectable (via syringe) implantation including at the wrist and with utility for all individuals of all skin tones would be immensely advantageous. In contrast to UV or blue light, red and NIR light penetrate deeper within tissues and in the context of a glucose sensor would allow the light source to be placed externally on the body, including on those with darker skin tones.^12^ In this scenario, sensors using red and NIR fluorescent probes could have electronic or optical components located in external devices and thus allow a small implant size.^13,14^ Our group has designed and created a highly sensitive fluorescent assay for glucose sensing using a novel competing ligand 8-aminopyrene-1,3,6-trisulfonic acid (APTS)-labeled mannotetraose (MT) and recognition molecule tetramethylrhodamine (TRITC)-labeled Concanavalin A (ConA).^15,16^ ConA is a tetrameric protein above pH 5.6 with four available sites that allow reversible binding of glucose or mannose.^17^ To prevent the common issue of ConA aggregation, we developed two successful approaches to address this obstacle: (1) a small-molecule fluorescent ligand with a single binding moiety to prevent a single ligand from binding to multiple ConA sites at once and (2) PEGylation of ConA to reduce aggregation of exposed hydrophobic regions of denatured ConA.^16,18^ Overall, this design resulted in a sensitive response to the physiological concentrations of glucose. However, the blue range excitation wavelength of APTS (450 nm) hinders the development of an implantable design with the light source located externally on the body.^15,19^

In this paper, red excitable fluorescent competing ligands were developed for a competitive binding, nonenzymatic, ConA-based glucose biosensor (Figure 1). We describe the synthesis, characterization, and binding affinity (to PEGylated-ConA) of three Cy5.5-labeled mannose-based molecules: mannotetraose, mannotriose, and mannobiose. Representing a robust synthetic strategy, a single fluorophore (Cy5.5) was conjugated to each fully preserved mannose molecule via reductive amination. This synthesis included the addition of an amino linker to first bind to the reducing terminal of mannose via reductive amination and second to the Cy5.5 fluorophore via an NHS ester. A glucose sensing was conducted *in vitro*. The polarization-based method of fluorescence anisotropy (FA) was used to characterize the competitive binding assay. A computational model was used in the optimization of concentration combinations of ConA with the competing ligand in competitive binding assays. The computational results were also evaluated by comparing them with the experimental observations.

**Figure 1:**
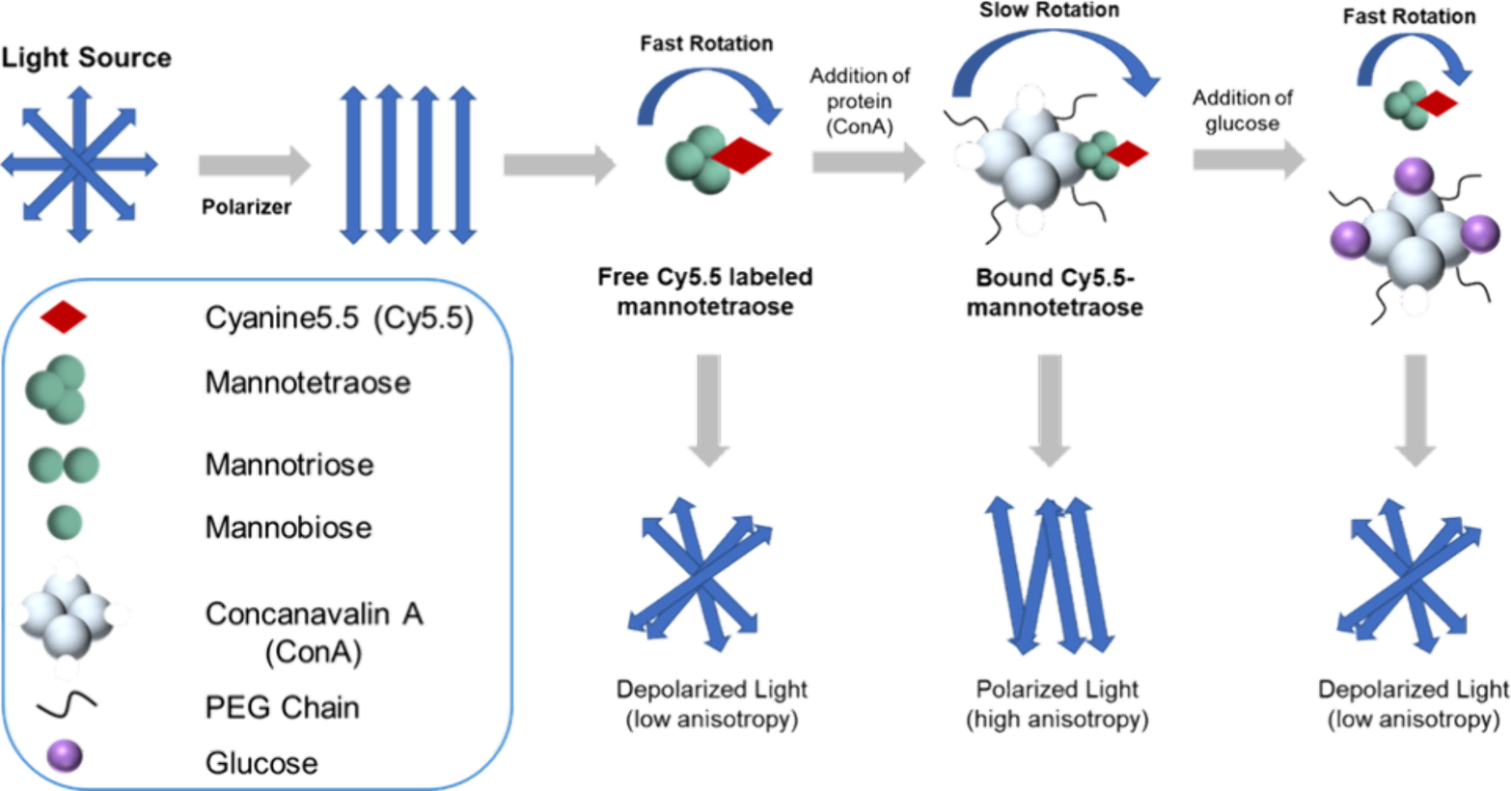
Schematic of a competitive binding ConA-based assay with the competing ligand Cy5.5-mannose-based molecules (shown here with mannotetraose) using fluorescence anisotropy as the transduction mechanism.

## Experimental Methods

### Materials and Reagents

Tris-buffered saline (TBS) tablets, sodium bicarbonate, sodium carbonate, 1-(*N*-Boc-aminomethyl)-4-(aminomethyl)benzene, dimethyl sulfoxide (DMSO), acetic acid, sodium cyanoborohydride (NaBH_3_CN), trifluoroacetic acid (TFA), triisopropylsilane (TIPS), diethyl ether, *N*,*N*-diisopropylethylamine (DIPEA), dimethylformamide (DMF), ConA Type IV lyophilized powder, methoxypolyethylene glycol 5000 propionic acid *N*-succinimidyl ester, methyl α-D-mannopyranoside (MaM), and Amicon Ultra-2 Centrifugal Filters Unit 30 kDa MWCO were purchased from Sigma (St. Louis, MO). Cyanine5.5 (Cy5.5) NHS ester was purchased from Lumiprobe (Hunt Valley, Maryland). The sugars α1-3,α1-6 mannotetraose, α1-3,α1-6 mannotriose, and α1-4 mannobiose were purchased from DextraUK (Reading, UK). The Tris buffer (pH 7.4), containing 50 mM Tris–HCl and 150 mM NaCl, and 0.1 M carbonate-bicarbonate buffer (pH 8.5) were prepared in deionized water.

### Synthesis and Purification of Cy5.5-mannose Conjugates

Three mannose variants (i.e., mannotetraose, mannotriose, and mannobiose) were chosen for Cy5.5 labeling. The reaction mixture consisted of 1 mol equiv of the mannose molecules, 10 mol equiv of 1-(*N*-Boc-aminomethyl)-4-(aminomethyl)benzene (a bifunctional linker containing a protected amine group), and 10 mol equiv of NaBH_3_CN in DMSO/acetic acid (7:3 v/v) in an oil bath at 36 °C for 5 days. The samples were then diluted with water such that the volume of DMSO was <1% to allow for drying via a LABCONCO FreeZone 2.5L benchtop freeze-dryer. The crude product was dissolved in water, and preparative HPLC was performed on a Shimadzu HPLC system using a preparative column (Shimadzu Shim-pack GIS C_18_, 20 × 250 mm, 10 μm) at a flow rate of 10 mL/min. A linear gradient of 0 to 60% solvent B over 15 min was used (solvent A: 0.1% TFA in water; solvent B: 0.1% TFA in acetonitrile) with detection at 220 nm. The product was confirmed via (+)ESI mass spectrometry using direct infusion into the mass spectrometer, a Q Exactive (QE) Focus (Thermo Scientific, Massachusetts, US).

The resulting pure mannose-linker samples were dried via lyophilization and dissolved in a mixture of TFA (95% v/v), TIPS (2.5% v/v), and water (2.5% v/v) and shaken for 2 h to remove the BOC (tert-butoxycarbonyl)-protecting group from the linker. Diethyl ether (at −20 °C) was slowly added to the sample solution, until precipitation was complete. Immediately, the precipitate was collected by centrifugation at 4 °C at 4000 rpm for 15 min and the supernatant was removed. The product was washed with water and freeze-dried before Cy5.5 labeling. The product was confirmed via (+)ESI with methods similar to those previously described.

With the removal of the BOC protecting group, the Cy5.5 NHS ester was attached to the free amine on the linker. The reaction mixture consisted of 1 mol equiv of mannose-linker, 2 mol equiv of Cy5.5 NHS ester, and 4 mol equiv of DIPEA in DMF shaken for 24 h at room temperature. Purification via HPLC was performed with the same system as described above. The linear gradient of 5 to 95% of solvent B over 25 min was used with the Cy5.5 dye detected at 600 nm. The product was confirmed using identical (+)ESI methods as previously described. Purity of the samples was verified via the LC-MS performed on a Thermo Scientific QE Focus using a semipreparative column (Thermo Scientific Acclaim 20 C18 2.1 × 150 mm). A linear gradient of 5 to 98% solvent B over 10 min was used (solvent A: 0.1% formic acid in water; solvent B: 0.1% formic acid in acetonitrile). The concentration of the final product was determined by measuring the peak absorbance via a Cary 300 UV–vis spectrophotometer at approximately 680 nm and using the extinction coefficient of Cy5.5 NHS Ester (198,000 M^–1^ cm^–1^).

### ConA PEGylation and Characterization

The primary amines of ConA were utilized for PEGylation purposes using a 5 kDa mPEG propionic acid NHS ester (mPEG-NHS). ConA was dissolved in a carbonate–bicarbonate buffer (10 mg/mL), and to protect its binding sites from potential PEGylation, MaM was added at 1.9 mg/mL. The solution was placed on a rocking platform shaker for at least 20 min to ensure optimal binding. Next, 32 mol equiv of mPEG-NHS was added per 1 mol equiv of ConA and placed again on the rocking platform shaker for 4 h at room temperature before being removed and allowed to further react at room temperature. The sample was then transferred at least 24 h later to Amicon Ultra-2 Centrifugal Filters (30 kDa MWCO, 2 mL volume) and washed seven times with either water or TBS (pH 7.4) at 3000 × *g* with 20 min runs at 4 °C using an Eppendorf Centrifuge 5810R. The concentration of the sample was calculated by measuring the peak absorbance (∼280 nm) on a Cary 300 UV–vis spectrophotometer and using the extinction coefficient of 118,560 M^–1^ cm^–1^.^18^ Monitoring of aggregation was conducted with dynamic light scattering (DLS) to measure the average particle size (Z-average) and polydispersity index (PDI) using a Zetasizer Nano (Malvern Instruments Ltd., Worcestershire, UK) based on methods provided by Locke.^18^

PEGylation was analyzed via matrix-assisted laser desorption ionization time-of-flight mass spectrometry (MALDI-TOF MS) performed using a Bruker Microflex MALDI-TOF mass spectrometer (Bruker Daltonics) operated using FlexControl software, version 3.4, under optimized conditions in positive linear mode. Sinapinic acid (3,5-dimethoxy-4-hydroxycinnamic acid) and trans-2-[3-(4-*tert*-butyl-phenyl)-2-methyl-2-propenylidene] malononitrile (DCTB) were used as a matrix. The sample and matrix were prepared at concentrations of 1 and 10 mg/mL respectively. The sample solution was mixed with the matrix in a volume ratio of 1:5. About 0.5 μL of this mixture was deposited on a stainless-steel sample holder. After being air-dried, the sample was analyzed using MALDI-TOF MS.

### Binding Studies

All equilibrium binding was conducted using a QuantaMaster spectrofluorometer (PTI) by measuring the fluorescence emission spectra of Cy5.5-labeled mannotetraose, mannotriose, and mannobiose with polarizers positioned in the path of the excitation and emission light. These FA measurements were collected by setting the polarizers in specified vertical (*V*) or horizontal (*H*) orientations, where vertical is defined as the direction perpendicular to the plane of the beam and horizontal is the direction perpendicular to *V*.^20^ The anisotropy (*r*) was calculated using the following equation (eq 1) where *I*_VH_ is fluorescent intensity with a vertical excitation polarizer and a horizontal emission polarizer, *I*_VV_ is fluorescent intensity with vertical excitation and emission polarizers, and *G* is the correction factor of the instrument (*G* = *I*_VH_/*I*_VV_).^21^
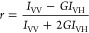
1

These fluorescence binding studies were performed in TRIS buffer (pH 7.4) with an excitation wavelength of 680 nm. Each cuvette contained a constant concentration of Cy5.5-labeled mannose (0.06 μM) and serial dilutions of either ConA or PEG-ConA were added, and time was given for equilibrium to be reached. The competitive binding model was used as a guide to select the optimal concentrations of PEG-ConA for 0.1 μM Cy5.5-labeled mannose conjugates, which would lead to the most sensitive response to glucose within the physiological glucose range. Glucose solutions were prepared in TBS to where a 1 mL volume of each solution was added to 1 mL of the doubly concentrated assay to produce final glucose concentrations ranging from 0 to 5000 mg/dL. The most sensitive assay design was then exposed to glucose concentrations within the physiological range (0–400 mg/dL) that had been quantified via a YSI biochemistry analyzer. The *r* values were then plotted versus the measured glucose concentration, and a best fit was found and used for glucose concentration prediction.

### Computational Model of Competitive Binding

With the dissociation constants determined experimentally via the binding affinity studies, a competitive binding model was utilized to calculate the optimal concentrations of ConA and mannotetraose, mannotriose, and mannobiose for high-sensitivity recognition within physiological glucose concentrations. The model was created in MATLAB using equations derived by Wang (eqs 2–6) to predict the percent of the competing ligand (Cy5.5 labeled mannose) unbound/free from ConA due to the introduction of glucose into the system.^22,23^ The following equations describe the competitive binding mechanism.
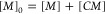
2
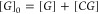
3
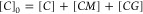
4
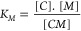
5
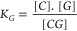
6Here [*M*], [*G*], and [*C*] represent the concentrations of the free/unbound Cy5.5 mannose conjugates, glucose, and ConA, respectively. The total concentrations present of each component in the competitive binding system are denoted as [*M*]_0_, [*G*]_0_, and [*C*]_0_, and ConA bound to the mannose sugars or glucose are defined as [*CM*] and [*CG*].^23^ The dissociation constant of mannose and ConA, *K_M_*, was calculated using results from the binding studies, and the dissociation constant of glucose and ConA, *K_G_*, has a known value of 2.5 × 10^–3^ M.^24^ In the binding studies, we assumed a ConA tetramer as one protein, and all dissociation constants used in this model are based on this assumption. Therefore, [*C*] represents the concentration of each ConA protein as a whole instead of four individual monomers.

By substituting eqs 5 and 6 into eq 4, a cubic equation with a variable [*C*] was obtained. Solving the cubic equation generated the solution of [*C*], which is the concentration of ConA unbound/free from mannose sugars or glucose. Using the obtained [*C*] value and applying eqs 2 and 5, we got the values of [*M*], which is the concentration of the mannose sugars unbound/free from ConA. The competitive binding was optimized with the goal of detecting glucose in the target physiological range of 0 to 400 mg/mL. To achieve this, the percentage of mannose sugars unbound/free from the ConA at the high concentration of glucose ([*G*]_0_ = 400 mg/mL) and the percentage of mannose sugars unbound/free from the ConA at the low concentration of glucose ([*G*]_0_ = 0 mg/mL) were calculated. The difference between the two percentages (eq 7) was used as the parameter to be optimized in the model, because maximizing this difference will lead to a wide dynamic range of the assay signal. In the computational model, the concentrations of mannose ([*M*]_0_) and ConA ([*C*]_0_) were varied to maximize the fraction difference. The model provided a guide for selecting the optimal concentration ratios of PEG-ConA and the Cy5.5 labeled mannose conjugates. The derivation of this computational model can be found in the Supporting Information.
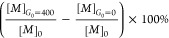
7

## Results and Discussion

### Fluorescent Competing Ligand Synthesis and Characterization

**Figure 2:**
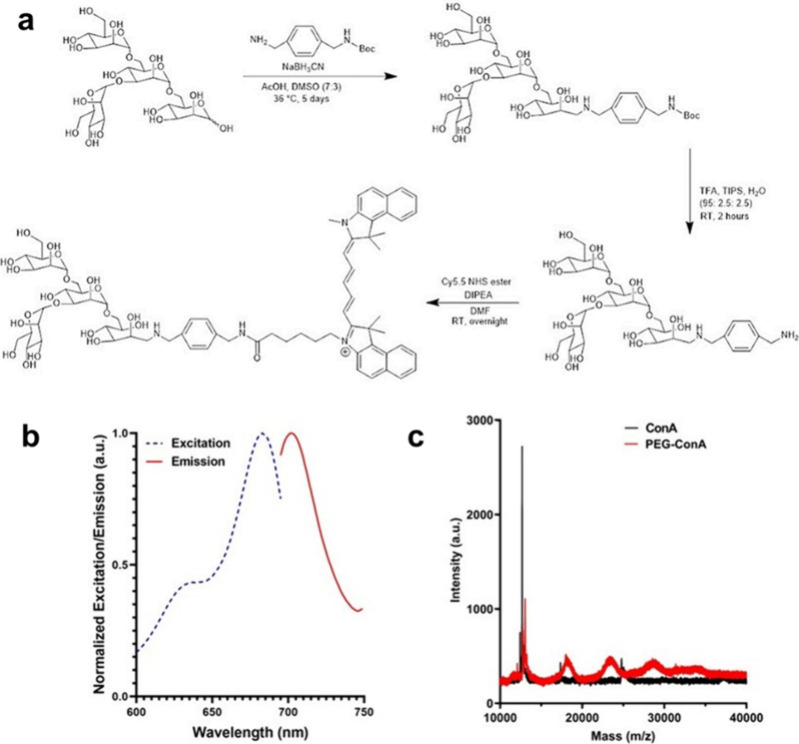
(a) Synthesis and (b) excitation and emission spectra of Cy5.5-mannotetraose. (c) Confirmation of PEGylation of ConA via MALDI-TOF.

A synthetic strategy was developed to prepare a fluorescently labeled competing ligand with a single fluorophore and to fully preserve mannose groups (Figure 2a). While the periodate oxidation method is commonly used to label glycans, it has commonly been found to destroy mannose groups, which are necessary for the binding to ConA.^25,26^ Reductive amination is another common method to label glycans, but would preserve the mannose groups.^27,28^ In our prior work, an amine-bearing fluorophore, APTS, was used to directly bind the fluorophore to the reducing terminus of the glycan via reductive amination. This one-step process was efficient but limited the options of fluorophores, especially those in the red or NIR range. Thus, this new method affords the ability to utilize a greater variety of commercially available fluorophores such as Cy5.5.

A small molecule, 1-(*N*-Boc-aminomethyl)-4-(aminomethyl)benzene, was selected as the linker to ensure a short distance between the mannose and the fluorophore. The short distance is preferred to enable a wide range of applications. For example, for a FRET (Förster resonance energy transfer) assay, the physical distance between the fluorophore pair is a significant factor for the efficiency of the energy transfer.^29^ In general, small molecules diffuse more quickly than large molecules. For the same mannose ligand that has a constant interaction surface, faster diffusion leads to stronger binding association.^30^ A shorter fluorescent molecule ensures no significant loss of FRET efficiency due to the increased separation of the donor and acceptor fluorophores. The synthesis maintains the reductive amination to bind the glycan to the linker with a free amine group. A second amine group is present on the linker molecule but is not free until a BOC deprotection step. The BOC protection is critical to avoid binding mannose molecules to both ends of the linker molecule during the first stage of the synthesis procedure, which would lead to a reduced yield of the intended final product. After the removal of the BOC protective group, a Cy5.5 NHS ester was bound to the free amine. For this study, Cy5.5 was used as the fluorophore for the ligand because of its far red excitation maximum of 683 nm.^31^ The developed method utilized the NHS ester of Cy 5.5 and can be easily generalized to other commercial dyes available as NHS esters. Using the developed method, Cy5.5-mannotetraose, Cy5.5-mannobiose, and Cy5.5-mannotriose were synthesized. The process was successful for all three small mannose molecules without any alterations to the synthesis or purification methods. The products were confirmed via the LC-MS, and UHPLC traces were collected (Figure S1) and (+)ESI-MS traces were analyzed (Table S1 and Figure S2). Chemical structures of all products can be found in Figure S2. For Cy5.5-mannotetraose, an excitation maximum was shown at 683 nm, and an emission maximum was shown at 702 nm through fluorescent characterization (Figure 2b). Additionally, the excitation and emission maximums were detected at 683 and 702 nm, respectively, for both Cy5.5-mannobiose and Cy5.5-mannotriose (Figure S3). The degree of PEGylation and the particle size of PEG-ConA were both determined to confirm successful binding of the PEG chains to the ConA in a comparable way to our previous work to prevent aggregation due to denaturation.^18^ Unmodified and PEGylated ConA were both analyzed via MALDI-TOF mass spectroscopy to confirm the binding of the PEG chains and provide an estimate of the number of chains bound. The unmodified ConA was seen at 12 689 Da, which represents approximately one-eighth of the tetrameric form of ConA (104,000 Da) (Figure 2c). With the binding of amine-reactive mPEG-SPA (5000 Da) to ConA, MALDI peaks were observed at 13,011 18,018, 23,472, 28,920, and 34,472 Da, therefore providing an estimate of zero to four conjugated PEG chains per one-eighth of ConA. The particle size of both the unmodified and PEGylated ConA was estimated using DLS. All DLS data was collected on unfiltered protein samples. ConA was dissolved in TBS (pH 7.4) and measured within 1 h of preparation to avoid aggregation. Two major peaks were detected at 9.00 ± 0.09 and 180.83 ± 41.95 nm with peak intensity percentages of 48.3 ± 2.4 and 40.0 ± 5.5, respectively. The estimated diameter of tetrameric ConA is 8 nm, which is comparable to the peak with the greatest intensity, and all greater diameters detected represent aggregates of the protein. The *z*-average particle size of PEG-ConA was 34.6 ± 0.3 nm, which was similar to the diameter found in our previous work of 30.0 ± 0.2 nm.^18^ The PDI represents the nonuniformity of particle size distribution on a scale from 0 to 1.^32^ The PDI for both unmodified and PEGylated ConA was 0.645 ± 0.020 and 0.265 ± 0.024, respectively, implying that ConA has a broader range of particle size than PEG-ConA, due to the formation of aggregates for unmodified ConA compared to PEG-ConA, as noted in our previous work.^18^

### Binding Studies of Cy5.5-Mannotetraose

**Figure 3:**
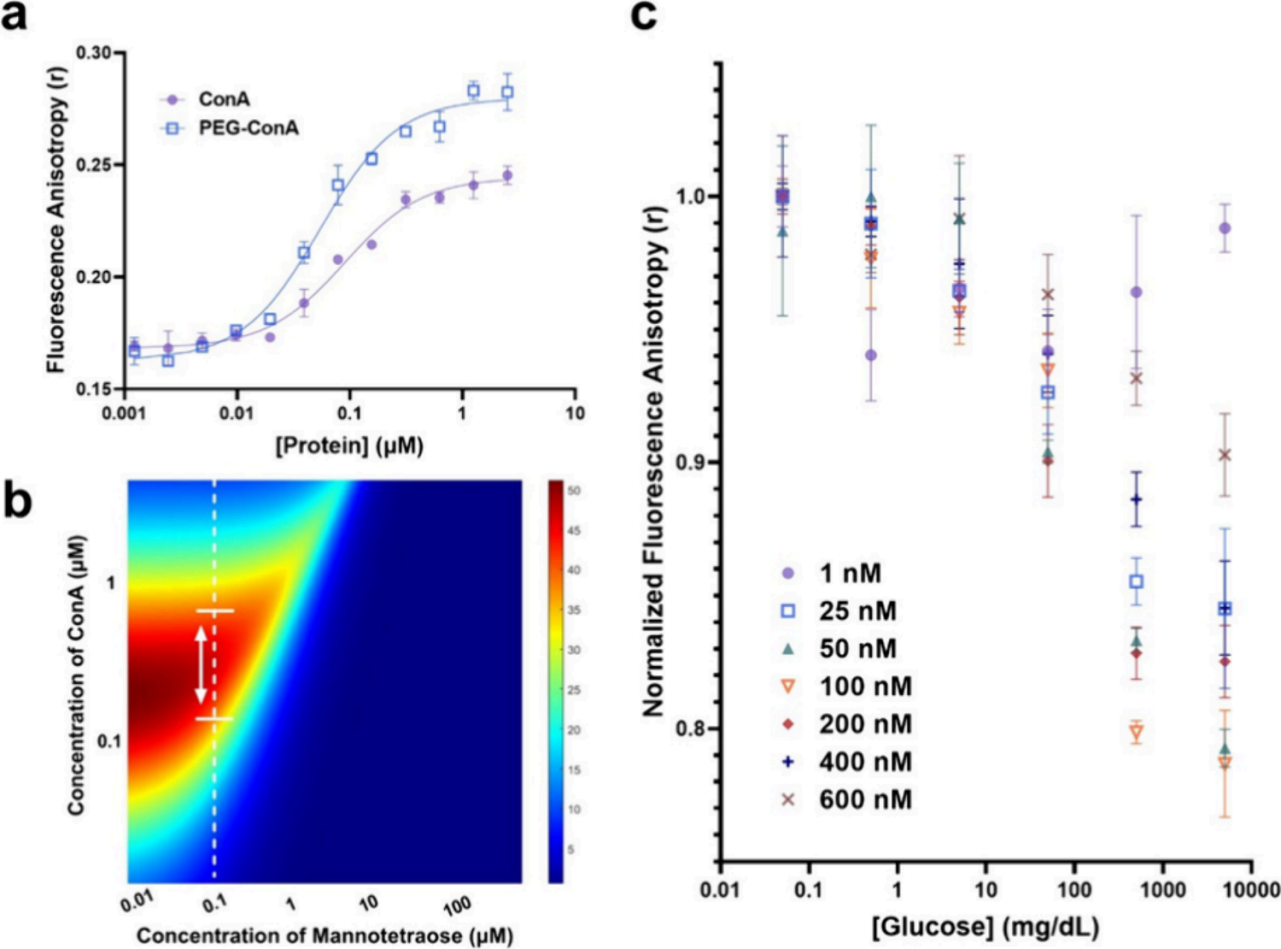
(a) Semilog plot and calibration fit of steady-state anisotropy measurements of 0.06 μM Cy5.5-mannotetraose with increasing concentrations of unmodified or PEGylated ConA. The association constants of unmodified ConA and PEG-ConA are calculated to be 1.12 × 10^7^ and 1.85 × 10^7^ M^–1^, respectively. (b) Sensitivity map from the computational model. The red regions predict the highest percentage of free ligand (mannotetraose) in the presence of physiological glucose concentrations. (c) Steady-state anisotropy measurements of 0.1 μM Cy5.5-mannotetraose when paired with varying concentrations of PEG-ConA and then introduced to increasing concentrations of glucose.

The dissociation constants of Cy5.5-mannotetraose with both unmodified and PEGylated ConA were determined by using FA. Although not a method for glucose sensing *in vivo* due to light scatter of tissues, FA was useful for initial characterization of the competing ligands. As more protein is introduced to a steady concentration of the ligand, more binding instances are expected. The small molecule, Cy5.5-mannotetraose, is excited via polarized light, and due to rotational diffusion, depolarization occurs. As this ligand binds to the much larger protein, the rate at which it tumbles will decrease, resulting in less depolarization and higher anisotropy values. The FA values were plotted in a semilog plot versus the concentration of the protein and fit to a Boltzmann curve to calculate the dissociation constant, *K_d_*, value (Figure 3a). Using the relationship between the dissociation and association constants (*K_a_* = 1/*K_d_*), the association constants of Cy5.5-mannotetraose to ConA and PEG-ConA were 1.12 × 10^7^ and 1.85 × 10^7^ M^–1^ respectively. These values were greater than the previously reported values from APTS-labeled mannotetraose^23^ indicating the better binding affinity of Cy5.5-mannotetraose. Comparing the values of PEG-ConA and unmodified ConA, Cy5.5-mannotetraose had a higher binding affinity for PEG-ConA (Table S2). The difference in the binding affinity between ConA and PEG-ConA may be explained by aggregation of the protein.

The dissociation constant values of Cy5.5-mannotetraose and PEG-ConA (*K_M_* = 5.4 × 10^–8^ M) and glucose and ConA (*K_G_* = 2.5 × 10^–3^ M), target glucose range (0–400 mg/dL), and minimum concentrations of interest for mannotetraose and ConA (0.1 μM for each) were put into the model (Figure 3b). Based on the model’s prediction, for 0.1 μM Cy5.5-mannotetraose, the ConA concentration with the highest predicted percent of free competing ligand was 0.3 μM. The concentration range of ConA with predicted values greater than 40% was 0.125 to 0.600 μM. Experimentally, the concentration of PEG-ConA with the most sensitive response to glucose within the 50 to 500 mg/dL range was 0.1 μM (Table S3). This 0.1 μM PEG-ConA concentration was within the orange region (values > ∼35% as depicted in Table S4) of the model signifying a high predicted sensitivity (Figure 3c). Comparing the results from the computational model and the experiment, the model showed the ability to provide a range of values to guide the assay optimization.

**Figure 4:**
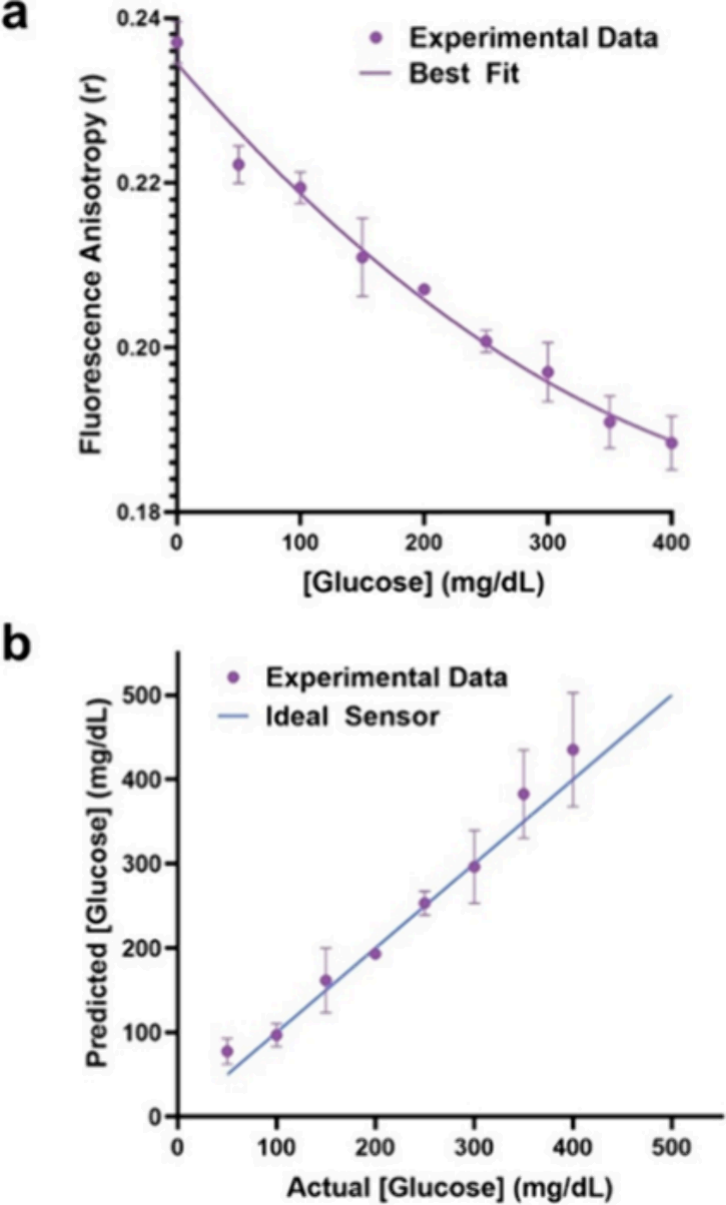
(a) Steady-state anisotropy of 0.1 μM Cy5.5-mannotetraose and 0.1 μM PEG-ConA in response to glucose within the physiological concentration range (0–400 mg/dL) with calibration fit. (b) Predicted versus actual glucose concentration of the response seen in panel (a) resulting in a percent MARD of 15.4% in the range of 50 to 400 mg/dL.

The competitive binding assay using the optimal concentration ratios of PEG-ConA and the Cy5.5-mannotetraose was then compared with an ideal sensor (YSI 290 Biochemistry Analyzer). Using the assay of 0.1 μM Cy5.5-mannotetraose and PEG-ConA, a sensitive response of the assay was observed for various glucose concentrations within the physiological range (Figure 4a). A line of best fit was calculated, used to predict the glucose concentrations, and compared to an ideal sensor. With a YSI 290 Biochemistry Analyzer, actual glucose concentrations were determined and plotted against the predicted values to calculate a standard error of calibration of 24.7 mg/dL and mean absolute relative difference (MARD) of 15.4% within the glucose concentration range of 50 to 400 mg/dL (Figure 4b). These results confirmed the reasonably good accuracy of the developed competitive binding assay in response to glucose. Further, ConA shows little to no binding affinity for other sugars, such as galactose, due to their structural differences. This affinity to the glucose has been shown experimentally in our previous work for APTS-mannotetraose, while the galactose shows no affinity to ConA.^33^

### Binding Studies of Cy5.5-Mannotriose and Cy5.5-Mannobiose

**Figure 5:**
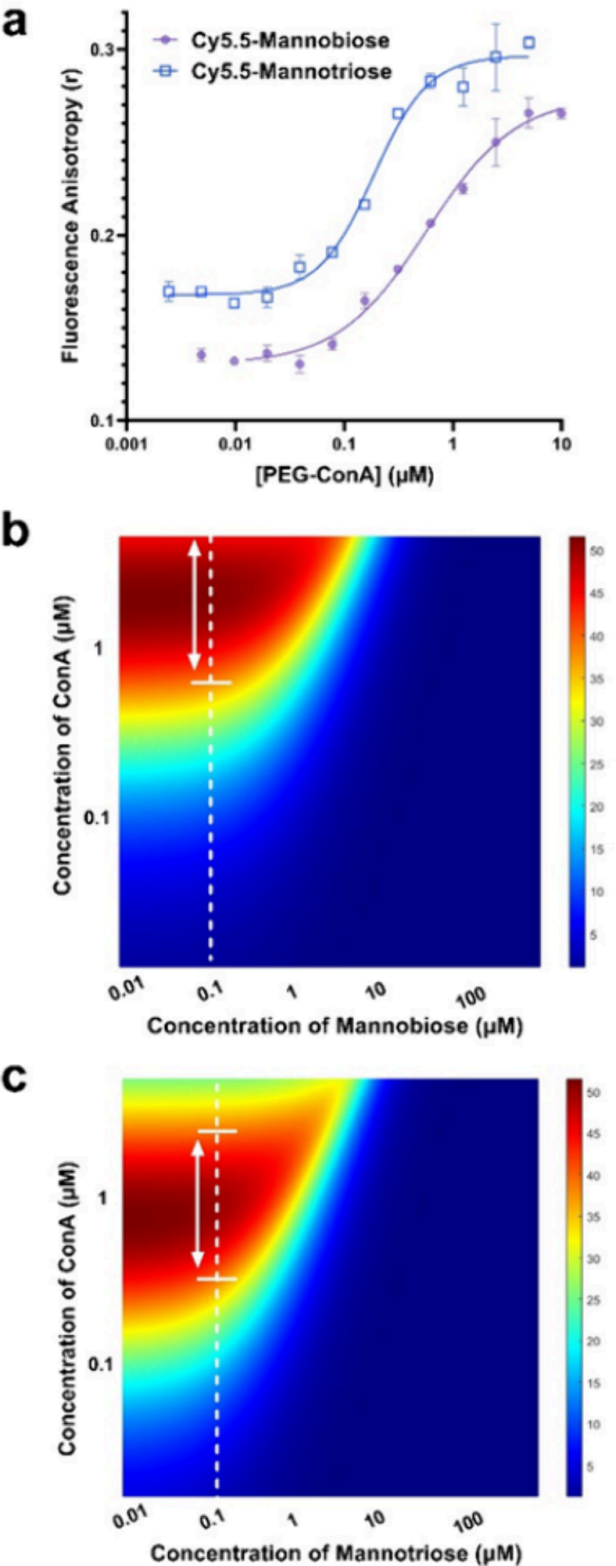
(a) Semilog plot and calibration fit of steady-state anisotropy measurements of 0.06 μM Cy5.5-mannobiose and Cy5.5-mannotriose with increasing concentrations of PEG-ConA resulting in association constants of 1.73 × 10^6^ and 5.33 × 10^6^ M^–1^ respectively. (b) Sensitivity maps of Cy5.5-mannobiose and (c) Cy5.5-mannotriose where the red regions represent the highest percent of free competing ligands in the presence of physiological glucose concentrations.

The synthesized Cy5.5-mannotriose and Cy5.5-mannobiose were evaluated and tested in a competitive binding glucose assay. FA measurements were collected to calculate the association constants of Cy5.5-mannobiose and Cy5.5-mannotriose with the PEG-ConA in a similar manner as with Cy5.5-mannotetraose (Figure 5a). The binding association constant values were found to be 1.73 × 10^6^ and 5.33 × 10^6^ M^–1^ for Cy5.5-mannobiose and Cy5.5-mannotriose, respectively. The binding association constant of Cy5.5-mannotriose and PEG-ConA was similar to the previously reported value (5.4 × 10^6^ M^–1^) of APTS-labeled mannotetraose and PEG-ConA.^23^ The Cy5.5-labeled mannose with the lowest binding affinity was found to be Cy5.5-mannobiose. The dissociation constants were used as input parameters for the competitive binding model and the concentrations of ConA with the greatest estimated percent free competing ligand within the physiological glucose range being 2.2 μM for 0.1 μM Cy5.5-mannobiose. Predicted values greater than 40% were within the ConA concentration range of 0.6 to 6 μM (Figure 5b). For 0.1 μM Cy5.5-mannotriose, the greatest estimated percent free competing ligand within the physiological glucose range was when paired with 0.75 μM ConA. Predicted values greater than 40% for Cy5.5-mannotriose were found for the ConA concentration range of 0.25 and 2.0 μM (Figure 5c).

**Figure 6:**
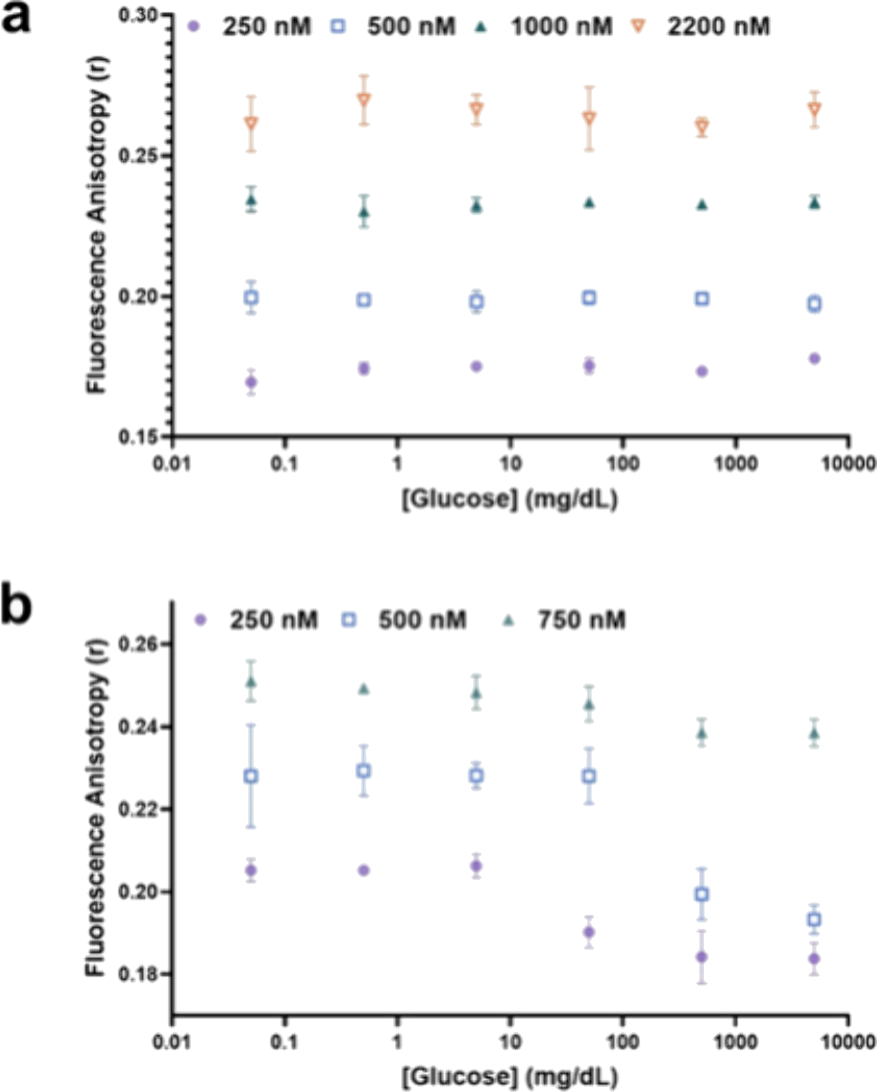
(a) Glucose response via steady-state anisotropy of 0.1 μM Cy5.5-mannobiose and (b) Cy5.5-mannotriose when paired with varying concentrations of PEG-ConA.

These concentration ratios were experimentally tested using the previously described method in the case of Cy5.5-mannotetraose. No FA changes were observed with changes to the concentration of glucose for 0.1 μM Cy5.5-mannobiose (Figure 6a), presumably due to the low binding affinity of Cy5.5-mannobiose and PEG-ConA making it a nonideal competing ligand in the physiological-range for glucose detection. For Cy5.5-mannotriose, 0.75 μM PEG-ConA lacked a sensitive response to glucose in the target range. Changes of FA from 5 to 500 mg/dL were similar for Cy5.5-mannotriose with 0.5 and 0.25 μM PEG-ConA; however, the assay with 0.5 μM PEG-ConA was seen to have the greatest sensitivity within the 50 to 500 mg/dL glucose concentration range (Figure 6b). For 0.25 μM PEG-ConA, the highest sensitivity appeared for glucose concentrations within the 5 to 50 mg/dL range, and because 0.5 μM PEG-ConA was within the “hot” region of Figure 5c (values >45%), the experimental results again showed the ability of the computational model to predict a range of values to guide assay optimization. It should be noted here that the parameters optimized in the computational model were based on the percent of free competing ligands at two extreme points (0 and 400 mg/mL glucose). To further hone the range, a modified parameter that splits the 0–400 mg/mL range into several segments, calculating the difference in each segment, and multiplying the differences together, could help to further improve the model by providing more specificity across each part of the physiological glucose range.

## Conclusions

In this paper, we have presented a successful synthesis of new red excitable small-molecule competing ligands for a ConA-based glucose sensing assay. The synthetic strategy afforded preparation of Cy5.5-mannotetraose, Cy5.5-mannobiose, and Cy5.5-mannotriose wherein a single fluorophore was conjugated to each fully preserved mannose molecule. The method utilized the NHS ester of Cy 5.5 and can be easily generalized to other commercial dyes bearing NHS esters. Of the three Cy5.5-labeled mannose conjugates synthesized, Cy5.5-mannotetraose demonstrated the most sensitive response to the physiological range of glucose, as evaluated by FA. Cy5.5-mannobiose showed no response, most likely due to its lower binding affinity to PEG-ConA. The computational model created for predicting the optimal concentration ratios of mannotetraose to ConA provided accurate guidance to the order of magnitude of interest. The sensing assay using the optimal concentration ratios of PEG-ConA and Cy5.5-mannotetraose showed good accuracy compared with an ideal sensor. FA is not intended to be the final transduction method for an implantable sensor due to tissue greatly depolarizing light. Rather, upon validation of this assay’s competitive binding capabilities, future steps will include modification of the assay using a fluorescence intensity-based transduction method like FRET and encapsulation of the assays into a biocompatible injectable housing. It is worth mentioning that the binding dissociation constants used in this work were measured from a standard buffer. Measuring these constants using interstitial fluid from human subjects will be necessary for a more accurate modeling of the assay for in vivo measurements.

## Supporting Information Available

UHPLC traces of the final products Cy5.5-mannobiose, Cy5.5-mannotriose, and Cy5.5-mannotetraose (Figure S1); chemical structures and ESI-MS traces for Cy5.5-mannobiose, Cy5.5-mannotriose, and Cy5.5-mannotetraose; excitation and emission spectra (Figure S3); analytical data (UHPLC-ESI-MS) for each Cy5.5-mannose compound (Table S1); comparison of the association constants of Cy5.5-mannotetraose to unmodified ConA and PEG-ConA with 95% confidence interval (CI) (Table S2); comparison of FA values for 100 nM Cy5.5-mannotetraose paired with varying concentrations of PEG-ConA after exposure to glucose of concentrations 50 and 500 mg/dL (Table S3); Comparison of the differences between the percentages of mannose sugars unbound/free from the ConA at the lowest (0 mg/dL) and the greatest (400 mg/dL) glucose concentrations (Table S4) (PDF)
