# Supplementary material for: Computational Model-Assisted Development of a Nonenzymatic Fluorescent Glucose-Sensing Assay
Source: ACS Sens. 2024 Nov 13;9(11):6218–27. doi: 10.1021/acssensors.4c02117 (PMC11590106; doi:10.1021/acssensors.4c02117)
Supplement: Supplementary file 1 — se4c02117_si_001.pdf [file se4c02117_si_001.pdf]

SUPPORTING INFORMATION

Computational Model-Assisted Development of a Non-enzymatic Fluorescent Glucose Sensing Assay

Lydia Colvin[a], Diana Al Husseini[a,d,*], Dandan Tu[a,d], Darin Dunlap[a], Tyler Lalonde[b], Muhammed Üçüncü[c], Alicia Megia-Fernandez[c], Mark Bradley[c], Wenshe Liu[b], Melissa A. Grunlan[a], Gerard L. Coté[a,d,e]

[a]Department of Biomedical Engineering, Texas A&M University, College Station, TX 77843, USA

[b]Department of Chemistry, Texas A&M University, College Station, TX 77843, USA

[c]School of Chemistry, University of Edinburgh, Edinburgh EH9 3FJ, UK

[d]Center for Remote Health Technologies and Systems, Texas A&M Engineering Experiment Station, College Station TX 77843, USA

[e]Department of Electrical and Computer Engineering, Texas A&M University, College Station, TX 77843, USA

Corresponding Author
**Email:* dianaalhusseini1@tamu.edu

Derivation of Computational Model of Competitive Binding

The competitive binding process can be described by:
$C + M \rightleftharpoons CM, G + C \rightleftharpoons CG$ (1)

$$K_M = \frac{[C]\cdot[M]}{[CM]} \quad (2)$$

$$K_G = \frac{[C]\cdot[G]}{[CG]} \quad (3)$$

Here M represents free Cy5.5 mannose conjugate, G represents free glucose, and C represent free ConA. CM represents the ConA bound with mannose conjugate. CG represents the ConA bound with glucose. K_G is the dissociation constant of glucose and ConA. K_M is the dissociation constant of mannose and ConA. Conservation of mass requires that:

$$[M]_0=[M]+[CM] \quad (4)$$

$$[G]_0 = [G] + [CG] \quad (5)$$

$$[C]_0 = [C] + [CM] + [CG] \quad (6)$$

Here [M], [G], and [C] represent the concentrations of the free/unbound Cy5.5 mannose, glucose, and ConA respectively. The total concentrations present of each component of the competitive binding system are denoted as $[M]_0$, $[G]_0$, and $[C]_0$. The concentration of ConA bound to the mannose or glucose are denoted as [CM] and [CG], respectively.

Substitution of [M] in equation (2) using equation (4):

$$K_M = \frac{[C]\cdot([M]_0 - [CM])}{[CM]}$$

$$K_M \cdot [CM] = [C]\cdot([M]_0 - [CM])$$

$$(K_M + [C])\cdot[CM] = [C]\cdot[M]_0$$

$$[CM] = \frac{[C][M]_0}{K_M + [C]} \quad (7)$$

Similarly, substitution of [G] in equation (3) using equation (5):

$$K_G = \frac{[C]\cdot([G]_0 - [CG])}{[CG]}$$

$$K_G \cdot [CG] = [C]\cdot([G]_0 - [CG])$$

$$(K_G + [C])\cdot[CG] = [C]\cdot[G]_0$$

$$[CG] = \frac{[C][G]_0}{K_G + [C]} \quad (8)$$

Substitution of [CG] and [CM] in equation (6) using equation (7) and (8):

$$[\mathrm{C}]_0 = [\mathrm{C}] + \frac{[C][M]_0}{K_M + [C]} + \frac{[C][G]_0}{K_G + [C]}$$

$$[\mathrm{C}]_0 \cdot (K_M + [C]) \cdot (K_G + [C]) = [\mathrm{C}] \cdot (K_M + [C]) \cdot (K_G + [C]) + [C][M]_0 \cdot (K_G + [C]) + [C][G]_0 \cdot (K_M + [C])$$

The above equation can be rearranged and expressed as:

$$[\mathrm{C}]^3 + x[\mathrm{C}]^2 + y[\mathrm{C}] + z = 0 \quad (9)$$

where

$\mathrm{x} = K_G + K_M + [\mathrm{G}]_0 + [\mathrm{M}]_0 - [\mathrm{C}]_0$

$y = K_M([\mathrm{G}]_0 - [\mathrm{C}]_0) + K_G([\mathrm{M}]_0 - [\mathrm{C}]_0) + K_G K_M$

$\mathrm{z} = -K_G K_M [\mathrm{C}]_0$

According to the method outlined by Wang[1] and the physical condition that the [C] value should be ≥ 0, the root of equation (9) can be expressed as:

$$[C] = -\frac{x}{3} + \frac{2}{3}\sqrt{(x^2 - 3y)}\, cos\frac{\theta}{3} \quad (10)$$

where

$$\theta = arccos \frac{-2x^3 + 9xy - 27z}{2\sqrt{(x^2 - 3y)^3}}$$

Substitution of [CM] in equation (4) using equation (7):

$$[\mathrm{M}]_0 = [\mathrm{M}] + \frac{[C][M]_0}{K_M + [C]}$$

$$[\mathrm{M}] = [\mathrm{M}]_0 - \frac{[C][M]_0}{K_M + [C]}$$

$$(K_M + [C])[\mathrm{M}] = K_M [\mathrm{M}]_0$$

$$[\mathrm{M}] = \frac{K_M [\mathrm{M}]_0}{(K_M + [C])} \quad (11)$$

In this study, the K_G is 2.5×10^{-3} M. The K_M is 5.4×10^{-8} M for Cy5.5-mannotetraose and PEG-ConA. Given a specific $[M]_0$, $[G]_0$, and $[C]_0$, the [C] value can be calculated using equation (10). The calculated [C] value can be used in equation (11) to calculate [M], which is the concentration of free mannose after the assay reaches a binding equilibrium.

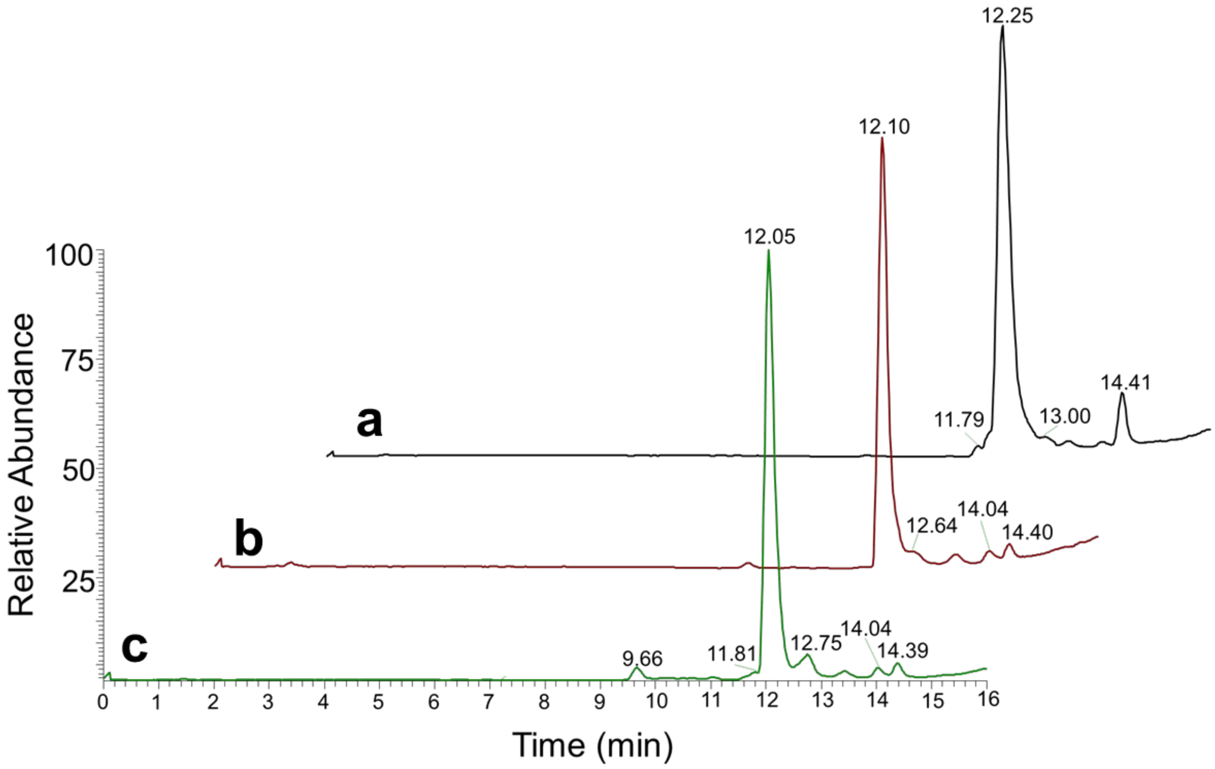

Supporting Figure S1. UHPLC traces of the final products Cy5.5-mannobiose (A), Cy5.5-mannotriose (B), and Cy5.5-mannotetraose (C).

Supporting Table S1. Analytical data (UHPLC-ESI-MS) for each Cy5.5-mannose compound

**Compound Name**	**Calculated Mass**	**Found Mass**
Cy5.5-mannobiose	1027.5427 ($[M+H])^+$)	1027.5415 ($[M+H])^+$)
Cy5.5-mannotriose	1189.5955 ($[M]^+$)	1189.5636 ($[M]^+$)
Cy5.5-mannotetraose	1351.6483 ($[M+H])^+$)	1351.6466 ($[M+H])^+$)

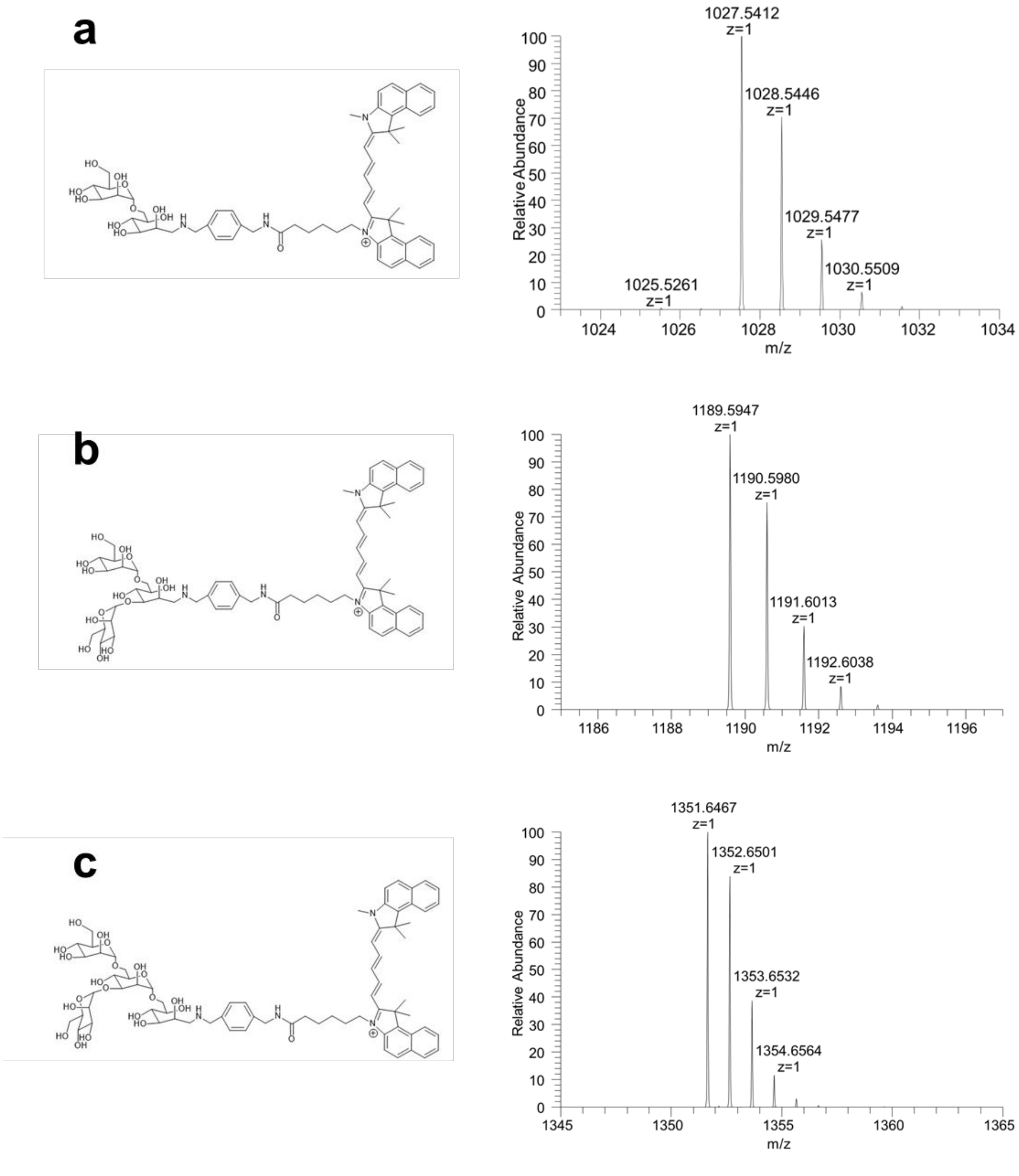

Supporting Figure S2. Chemical Structures and ESI-MS Traces for (a) Cy5.5-mannobiose, (b) Cy5.5-mannotriose, and (c) Cy5.5-mannotetraose.

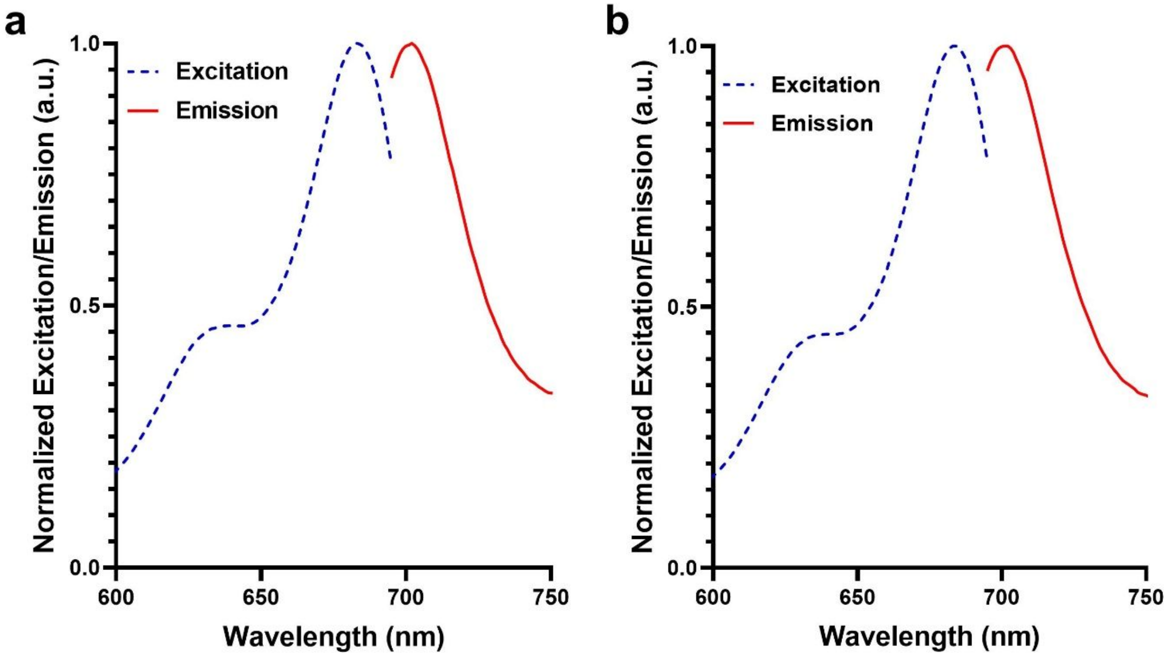

Supporting Figure S3. Excitation and Emission Spectra of (a) Cy5.5-mannobiose and (b) Cy5.5-mannotriose, the peak excitation was at 683 nm and the peak emission was at 702 nm.

Supporting Table S2. Comparison of the association constants of Cy5.5-mannotetraose to unmodified ConA and PEG-ConA with 95% confidence interval (CI).

Protein	K_a (M^{-1})	95% CI (M^{-1})
Unmodified ConA	1.12 x 10^7	8.61 x 10^6 to 1.60 x 10^7
PEG-ConA	1.85 x 10^7	1.44 x 10^7 to 2.60 x 10^7

Supporting Table S3. Comparison of FA values for 100 nM Cy5.5-mannotetraose paired with varying concentrations of PEG-ConA after exposure to glucose of concentrations 50 and 500 mg/dL.

[PEG-ConA] (nM)	FA at 50 mg/dL (FA_{50})	FA at 500 mg/dL (FA_{500})	FA_{50} – FA_{500}
1	0.1565	0.1602	-0.0037
25	0.1760	0.1625	0.0135
50	0.1789	0.1649	0.0140
100	0.1865	0.1593	0.0272
200	0.1899	0.1746	0.0153
400	0.2082	0.1962	0.0120
600	0.2412	0.2333	0.0079

Supporting Table S4. Comparison of the differences between the percentages of mannose sugars unbound/free from the ConA at the lowest (0 mg/dL) and the greatest (400 mg/dL) glucose concentrations (Δ Percentages) calculated via eq. 7 within the computational model for 100 nM Cy5.5-mannotetraose paired with varying concentrations of PEG-ConA.

[PEG-ConA] (nM)	Δ Percentages (%)
1	0.49
25	11.44
50	21.07
100	34.78
200	45.80
400	45.06
600	39.67

References:

(1) Wang, Z.-X. An exact mathematical expression for describing competitive binding of two different ligands to a protein molecule. *FEBS letters* **1995**, *360* (2), 111-114.
